# *In vitro* selectivity, *in vivo* biodistribution and tumour uptake of annexin V radiolabelled with a positron emitting radioisotope

**DOI:** 10.1038/sj.bjc.6601262

**Published:** 2003-09-30

**Authors:** D R Collingridge, M Glaser, S Osman, H Barthel, O C Hutchinson, S K Luthra, F Brady, L Bouchier-Hayes, S J Martin, P Workman, P Price, E O Aboagye

**Affiliations:** 1 Cancer Research UK PET Oncology Group, Department of Cancer Medicine, Imperial College of Science Technology and Medicine, MRC Cyclotron Building, Hammersmith Hospital, Du Cane Road, London W12 0NN, UK; 2 Imaging Research Solutions Limited, MRC Cyclotron Building, Hammersmith Hospital, Du Cane Road, London W12 0NN, UK; 3 Department of Nuclear Medicine, University of Leipzig, Liebigstrasse 20A, 04103 Leipzig, Germany; 4 The Smurfit Institute of Genetics, Trinity College, Dublin, Ireland; 5 Cancer Research UK Centre for Cancer Therapeutics, Institute of Cancer Research, Sutton, Surrey SM2 SNG, UK

**Keywords:** apoptosis, annexin V, phosphatidylserine, PET imaging, TUNEL

## Abstract

The availability of a noninvasive method to detect and quantify apoptosis in tumours will enable tumour response to several cancer therapies to be assessed. We have synthesised two radiotracers, annexin V and the *N*-succinimidyl-3-iodobenzoic acid (SIB) derivative of annexin V, labelled with radio-iodine (^124^I and ^125^I) and provided proof of the concept by assessing specific binding and biodistribution of these probes to apoptotic cells and tumours. We have also assessed the tumour uptake of [^124^I]annexin V in a mouse model of apoptosis. RIF-1 cells induced to undergo apoptosis *in vitro* showed a drug concentration-dependent increased binding of [^125^I]annexin V and [^125^I]SIB–annexin V. In the same model system, there was an increase in terminal deoxynucleotidyl transferase-mediated nick end labelling (TUNEL)-positive cells and a decrease in clonogenic survival. Radiotracer binding was completely inhibited by preincubation with unlabelled annexin V. In RIF-1 tumour-bearing mice, rapid distribution of [^125^I]SIB–annexin V-derived radioactivity to kidneys was observed and the radiotracer accumulated in urine. The binding of [^125^I]SIB–annexin V to RIF-1 tumours increased by 2.3-fold at 48 h after a single intraperitoneal injection of 5-fluorouracil (165 mg kg^−1^ body weight), compared to a 4.4-fold increase in TUNEL-positive cells measured by immunostaining. Positron emission tomography images with both radiotracers demonstrated intense localisation in the kidneys and bladder. Unlike [^124^I]SIB–annexin V, [^124^I]annexin V also showed localisation in the thyroid region presumably due to deiodination of the radiolabel. [^124^I]SIB–annexin V is an attractive candidate for *in vivo* imaging of apoptosis by PET.

There is an increasing need for the development of assays to determine the pharmacodynamic effects of new cancer therapeutics (Workman, 2002). Since assays that require tumour samples have a number of limitations, development of less invasive imaging technologies is extremely important (Workman, 2002). Evasion of programmed cell death (apoptosis) is a phenotypic hallmark that is shared by most human tumours (Hanahan and Weinberg, 2000). Pathways leading to apoptosis are now considered valid targets for cancer chemotherapy (Sellers and Fisher, 1999). Apoptosis is also the outcome of treatment with radiation and several chemotherapy agents and, therefore, a noninvasive method of quantifying changes in apoptosis will be an attractive pharmacodynamic end point for anticancer therapies. Such pharmacodynamic end points are critical in the development of new molecular therapeutics (Workman, 2002). Beyond oncology, the methodology may potentially be applicable in neurodegenerative diseases, transplant rejection and autoimmune disease.

Externalisation of phosphatidylserine (PS) is a general feature of apoptosis. During apoptosis, deactivation of the enzymes translocase and floppase, which maintain PS in the inner leaflet of the plasma membrane, together with activation of the enzyme scramblase, result in the redistribution of PS on the outer surface of the membrane (Martin *et al*, 1995; Zwaal and Schroit, 1997). These processes occur prior to membrane blebbing and DNA degradation. Annexin-V, a 35 kDa calcium-dependent protein, binds with high affinity (*K*=10^−9^ M) specifically to PS residues that are displayed on the outer surface of apoptotic cells (Martin *et al*, 1995). This means that in the intact animal or patient, apoptosis can be detected by imaging the binding of radiolabelled annexin V to PS. Using single-photon emission computed tomography (SPECT), a number of research groups have imaged PS expression with [^99m^Tc]-radiolabelled annexin V during cardiac allograft rejection, fas-mediated fulminant hepatic apoptosis and in a murine model of cyclophosphamide-induced apoptosis (Blankenberg *et al*, 1998, 1999, 2001; Narula *et al*, 2001; Mochizuki *et al*, 2003).

The levels of apoptosis induced in the above models are high in comparison to what is usually achievable in animal and human solid tumours following chemotherapy or biological therapy (Aboagye *et al*, 1998; David *et al*, 1999; Griffon-Etienne *et al*, 1999; Herbst *et al*, 2002). We have developed positron-labelled annexin V that can ultimately be used for imaging apoptosis in tumours by positron emission tomography (PET). In theory, PET should provide higher sensitivity and resolution for imaging apoptosis than SPECT, although the latter is more widely available. The objective of the present study was to determine the feasibility of using iodinated-annexin V to detect apoptosis in tumours and to validate the methodology by comparison to classical measures of apoptosis *in vitro* and *in vivo*. Annexin V can be radiolabelled with ^125^I directly, to give [^125^I]annexin V, or indirectly, to give the *N*-succinimidyl iodobenzoic acid derivative ([^125^I]SIB–annexin V). We investigated both nonisotopically labelled peptides, comparing specific binding to PS and deiodination of the radiolabel *in vivo*. The limitations of using radiolabelled annexin V to detect apoptosis, including the fact that it binds to both apoptotic and necrotic cells, are discussed.

## MATERIALS AND METHODS

### Radiolabelling of annexin V

Polyhistidine-tagged annexin V was prepared according to standard molecular biology procedures. Briefly, the coding region of annexin V was amplified from a Jurkat cDNA library using gene-specific primers and was inserted into pProEXHTb by *Bam*HI/*Eco*RI digestion. Polyhistidine-tagged annexin V was induced in *Escherichia coli*, DH5*α* strain, and subsequently purified from bacterial lysates by incubation with nickel-nitrilotriacetic acid (Ni-NTA) resin. The purified annexin-V protein was eluted from the Ni-NTA resin in the presence of 100 mM imidazole. Imidazole was subsequently removed by extensive washing in annexin V binding buffer (1.8 mM CaCl_2_, 10 mM HEPES-NaOH pH 7.4, 150 mM NaCl, 5 mM KCl and 1 mM MgCl_2_). The polyhistidine-tagged annexin V was shown to be active by a modified competition assay using a fluorescene isothiocyanate (FITC)-annexin V kit (BD PharMingen, San Diego, CA, USA). The annexin V was directly radiolabelled with either iodine-125 (for *in vitro* and biodistribution studies) or ^124^I (for PET imaging studies) as previously described (Glaser *et al*, 2003). [^125^I]SIB and [^124^I]SIB were synthesised according to published methods (Garg *et al*, 1989; Koziorowski *et al*, 1998; Glaser *et al*, 2003) and purified by normal-phase high-performance liquid chromatography (HPLC). Labelling of annexin V with [^125^I]SIB/[^124^I]SIB was performed according to the method of Glaser *et al* (2003). All iodinated-proteins ([^125^I]annexin V, [^125^I]SIB–annexin V, [^124^I]annexin V and [^124^I]SIB–annexin V) were purified on a PD10 column. The radiochemical purity of effluents was determined by instant thin-layer chromatography and sodium dodecyl sulphate–polyacrylamide gel electrophoresis. Protein concentrations for the determination of pseudo-specific radioactivity were measured by the BCA assay (Peribo Science, Chester, UK).

### Binding of radiolabelled annexin V to apoptotic cells *in vitro*

The biological activity of radiolabelled annexin V was evaluated in exponentially growing RIF-1 fibrosarcoma (adherent) cells treated with 5-fluorouracil (5-FU) at 3 and 30 *μ*M for 24 h (5% CO_2_ incubator at 37°C). Control (phosphate-buffered saline; PBS) and 5-FU-treated cells were washed twice with PBS and 5 ml of annexin V binding buffer added. To determine specific binding, some cells were preincubated for 15 min with 100 × unlabelled annexin V before the addition of radiolabelled annexin. Cells were incubated with either [^125^I]annexin V (20–25 *μ*Ci) or [^125^I]SIB–annexin V (3–4 *μ*Ci) on a Gyro rocker at room temperature for 1 h. After incubation, cells were scraped and centrifuged (5000 r.p.m. for 5 min at 4°C) to obtain the pellet. The pellet was washed with PBS (5 ml), centrifuged and the resulting pellet was dissolved in 0.6 ml of 1% Triton X. Aliquots of this solution were used for assessment of protein content as described above. Aliquots were also counted on a *γ*-counter (Packard, Pangbourne, UK) to determine the percentage of radioactivity in labelled cells, that is, %bound radioactivity=[100 × bound radioactivity/(total radioactivity × protein content)]. All experiments were performed in triplicate and repeated three times.

### Determination of apoptosis by flow cytometry

The extent of apoptosis in the model systems described was evaluated by flow cytometry. A hallmark of apoptosis is extensive degradation of chromosomal DNA into oligomers of ∼180 base pairs producing large numbers of free 3′-hydroxy deoxynucleotide ends. The terminal deoxynucleotidyl transferase-mediated nick end labelling (TUNEL) assay measures these 3′-hydroxy deoxynucleotide ends (Gavrieli *et al*, 1992). To perform this assay, RIF-1 cells were treated as above and fixed in PBS containing 1% (w v^−1^) paraformaldehyde (methanol-free). Cells were incubated in 70% ethanol on ice for 30 min and stained using the ApoDirect TUNEL assay kit (PharMingen). The proportion of apoptotic cells was determined by flow cytometry (gated to exclude aneuploidy). Fluorescent activated cell sorting (FACS) analysis of FITC-stained cells was performed using the WinMDI software (FACS Care Facility, Scripps Research Institute, San Diego, CA, USA).

### Clonogenic survival

In addition to biochemical measures of apoptosis, the extent of radiolabelled annexin V labelling of RIF-1 cells was also compared to clonogenic survival, a ‘gold standard’ of cell death. Control (PBS) and 5-FU-treated (3 or 30 *μ*M for 24 h) cells were harvested by trypsinisation and diluted in serum-enriched media (1000, 3500 cells ml^−1^ for control; 1000, 3500, 10 000 cells ml^−1^ for 3 *μ*M; and 7000, 10 000 cells ml^−1^ for 30 *μ*M). The cells (1 ml) were plated in Petri dishes and incubated for 7–10 days. The plates were stained with crystal violet (0.2% in 70% ethanol) and the resulting colonies were counted. The surviving fraction was determined by comparing the number of colonies in control cells to that of treated cells. Four plates were used for each dose level and the experiment was repeated three times.

### Biodistribution of radiolabelled annexin V

The biodistribution of [^125^I]SIB–annexin V was evaluated in C3H/Hej mice (Harlan, Oxfordshire, UK) bearing RIF-1 tumours. All animal work was performed by licensed investigators in accordance with the United Kingdom's ‘Guidance on the Operation of Animals (Scientific Procedures) Act 1986’ (HMSO, London, UK, 1990) and in full compliance with government regulations and UKCCCR guidelines on animal welfare (Workman *et al*, 1998). To obtain tumours, 5 × 10^5^ RIF-1 cells were injected into the rear dorsum subcutis of the mice. Tumours were selected for biodistribution studies when they had reached 5–8 mm in diameter (100–300 mg). Tumour-bearing mice were injected intravenously via the lateral tail vein with 0.1 ml of [^125^I]SIB–annexin V (2–3 *μ*Ci). At selected times after injection (2, 10, 30, 60 and 120 min), mice were killed by exsanguination via cardiac puncture under general anaesthesia (isofluorane inhalation). Aliquots of heparinised blood were rapidly centrifuged (2000 ***g*** for 5 min) to obtain plasma. The radioactivity contained in the tumour, liver, kidney, spleen, lungs, stomach, heart, small intestines, large intestines, brain, muscle, bone blood, plasma, faeces and urine was determined in a *γ*-counter (Packard) and expressed as a percentage of injected dose per gram of tissue (%ID g^−1^). A minimum of three mice were used for each time point.

### Uptake of radiolabelled annexin V by apoptotic tumours *in vivo*

To establish whether drug-induced apoptosis in tumours results in increased radiolabelled annexin uptake *in vivo*, the uptake of [^125^I]annexin V and [^125^I]SIB–annexin V was determined in mice treated with PBS (control) or 5-FU at a single intraperitoneal dose of 165 mg kg^−1^ (48 h prior to the radiotracer experiments). RIF-1-bearing mice were injected intravenously via the lateral vein with 0.1 ml of [^125^I]annexin V or [^125^I]SIB–annexin V (3–4 *μ*Ci). To determine the specificity of radiolabelled annexin V binding, a modified competition assay was performed in which a separate cohort of mice were injected intravenously with unlabelled annexin V at 1 h before the [^125^I]annexin V injection. The concentration of unlabelled annexin V in this case was 100-fold greater than that in the injectate of [^125^I]annexin V. At 60 min postinjection of radiolabelled annexin V, mice were killed by exsanguination via cardiac puncture under general anaesthesia. The radioactivity contained in the tumour was determined in a *γ*-counter and expressed as tumor : blood ratio. A minimum of three mice were used for each time point.

### Determination of apoptosis *in vivo* by immunohistochemistry

Similar to the *in vitro* studies, the extent of apoptosis in the *in vivo* model system described above was evaluated using a modification of the TUNEL immunohistochemistry method previously described by Aboagye *et al* (1998). Briefly, control (PBS) and 5-FU-treated (165 mg kg^−1^ intraperitoneally (i.p.); 48 h after treatment) RIF-1 tumours were excised and fixed in formal saline. The tumours were then embedded in paraffin for sectioning. Paraffin-embedded histological sections (5 *μ*m) were deparaffinised, treated with proteinase K and stained using the ApoDirect TUNEL assay kit (PharMingen). The sections were mounted in a medium containing propidium iodide (PI) (Sigma, Poole, Dorset, UK) and viewed by fluorescence microscopy (Olympus BX51, Olympus Optical, Tokyo, Japan) with a dual filter for FITC and PI. The number of apoptotic (green fluorescence) cells in each field of view (× 40 magnification; 0.55 mm^3^) was counted. Two slices from three tumours per treatment group were evaluated; 10 fields of view were randomly selected (in light microscopy mode) for analysis.

### Imaging of radiolabelled annexin V in mice by PET

Initial whole-body PET imaging with ^124^I radiolabelled annexin was performed to evaluate the imaging potential of the tracer in mice. RIF-1 tumour-bearing mice were treated with a single injection of 5-FU (165 mg kg^−1^ i.p.) to induce apoptosis and scanned 48 h after 5-FU treatment. For PET scanning, the tail veins of the mice were cannulated after induction of anaesthesia with isofluorane/N_2_O/O_2_. For each scan, a tumour-bearing animal was placed prone within a thermostatically controlled jig and positioned in the bore of the scanner. [^124^I]Annexin V or [^124^I]SIB–annexin V (10–40 *μ*Ci) prepared in PBS was injected via the tail cannula. Emission scans were acquired on a quad-HIDAC small animal scanner (Oxford Positron Systems, Oxfordshire, UK) in list-mode format. Animals were scanned for 1 h from the time of injection. The acquired list-mode data were sorted into 0.5 mm sinogram bins for image reconstruction (3D image pixel size – 0.5 × 0.5 × 0.5 mm). A single static frame from 30 to 60 min postinjection was obtained and visualised using the image analysis software, Analyze (version 4.0; Biomedical Imaging Resource, Mayo Clinic, Rochester, MN, USA).

### Statistical analysis

Statistical analyses were performed using GraphPad Prism, Version 2.0C (GraphPad Software Inc., San Diego, CA, USA). Errors were expressed as a standard error of the mean (s.e.m.). The significance of comparisons between two data sets was determined using Student's *t*-test for two independent populations. The significance of comparison between more than two data sets was determined using a one-way analysis of variance. *P*⩽0.05 was considered significant.

## RESULTS

### Radiochemical profile of iodinated annexin V

The radiochemical yield, pseudospecific radioactivity and radiochemical purity of [^124^I]annexin V were 22.3%, 14.5 GBq *μ*mol^−1^, and 97.7%, respectively. The radiochemical yield, pseudospecific radioactivity and radiochemical purity of [^124^I]SIB–annexin V were 25% (from [^124^I]SIB), 1.6 GBq *μ*mol^−1^ and 96.7%, respectively. The radiotracers were stable for up to 4 days without significant deiodination (Glaser *et al*, 2003).

### Radiolabelled annexin V binds to apoptotic cells *in vitro*

Figure 1

**Figure 1:**
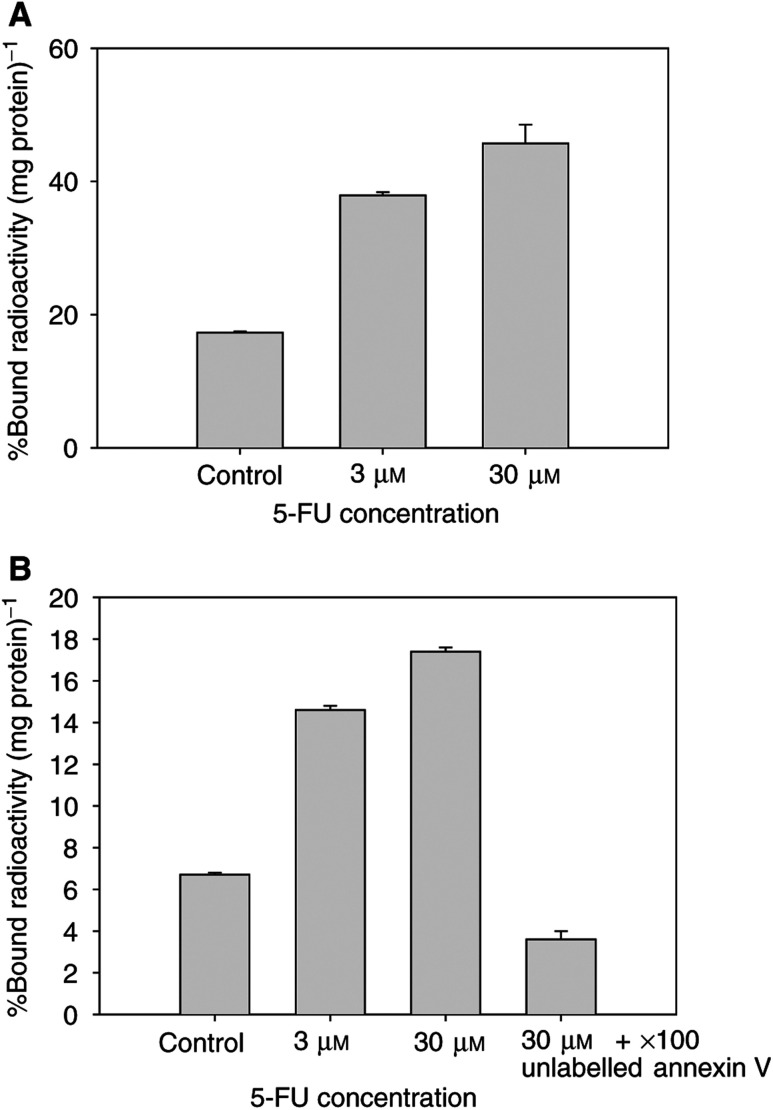
*In vitro* binding of radioiodinated annexin V to control (PBS) and 5-FU treated (3 and 30 *μ*M) RIF-1 cells. (**A**) [^125^I]SIB–annexin V and (**B**) [^125^I]annexin V. Data were expressed as %bound radioactivity (mg protein)^−1^. Data are mean±s.e.m. (*n*=9).

shows the binding of radiolabelled annexin V to RIF-1 cells. [^125^I]annexin V and [^125^I]SIB–annexin V both showed higher binding in treated cells compared to control cells (*P*=0.0001). The extent of binding was dependent on the concentration of 5-FU added. These results indicate that both methods of radiolabelling annexin V yield biologically active radiotracers. The proportion of radiotracer that was bound to apoptotic cells (%bound radioactivity (mg protein)^−1^) was, however, higher in the case of [^125^I]SIB–annexin V. A large decrease in radiotracer binding following pretreatment with unlabelled annexin V demonstrated the specificity of radiolabelled annexin V binding. It should be noted that radiotracer binding in this instance was lower than that of controls, indicating significant pretreatment levels of annexin-binding sites in control cells. Similar results were seen in HL60 cells treated with camptothecin at 5 *μ*M for 6 h (data not shown).

**Figure 2:**
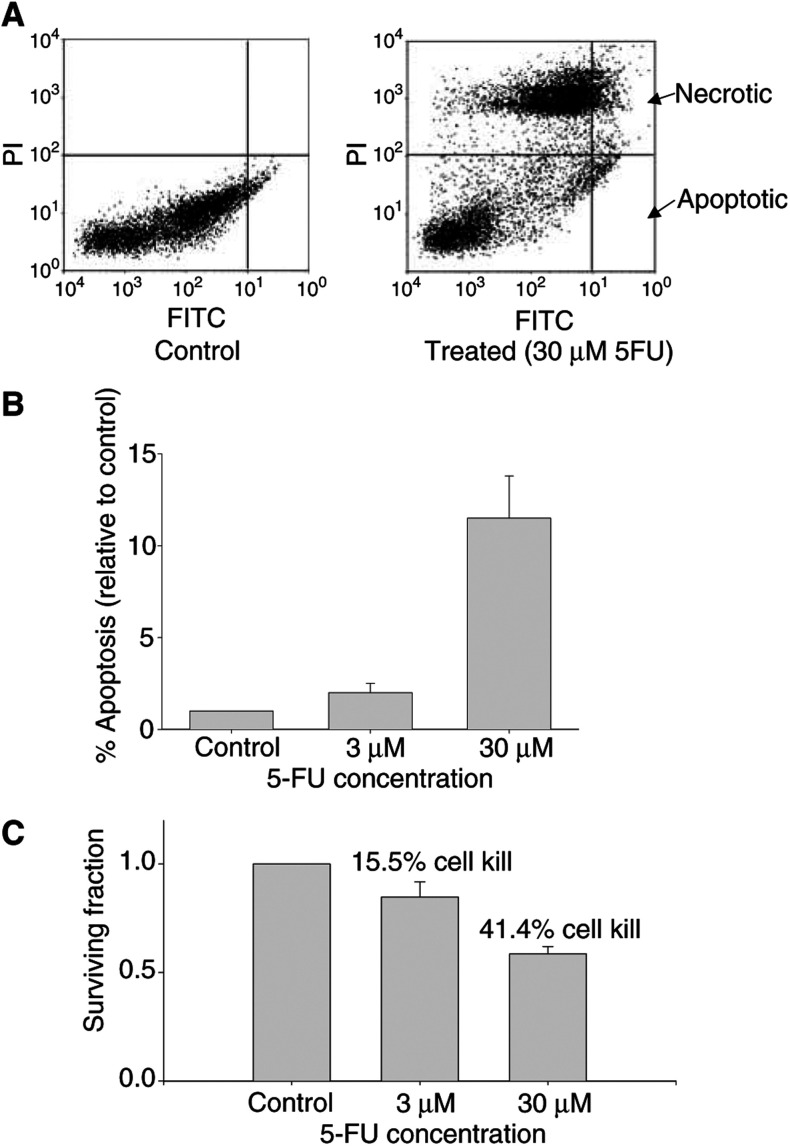
5-Fluorouracil-induced cell death in RIF-1 cells *in vitro*. (**A**) Flow cytometry of TUNEL-stained control (PBS treated) and 5-FU treated (30 *μ*M) cells. Data were analysed by setting quadrants on the FACS data such that 1% of control cells were in the lower right quadrant (FITC positive – PI negative; apoptotic cells); the same quadrant was then placed on the FACS data from corresponding treated cells. (**B**) Proportion of apoptotic cells relative to control cells. Data are mean±s.e.m. (*n*=4). (**C**) Clonogenic survival of control and 5-FU-treated cells expressed as a fraction of controls. Data are mean±s.e.m. (*n*=12).

The radiotracer binding data were compared to results obtained with TUNEL staining and clonogenic survival. Figure 2A

shows a typical flow cytometry output for control and 5-FU-treated cells. An increased proportion of apoptotic (FITC positive; PI negative) and necrotic (FITC positive; PI positive) cells was observed after 5-FU treatment. The percentage of TUNEL-positive apoptotic cells increased with drug treatment in a drug-concentration-dependent manner (*P*=0.0001; Figure 2B). In keeping with the drug-induced increase in apoptosis, there was a decrease in clonogenic survival of 15.5 and 41.4% (*P*=0.0001) at 5-FU concentrations of 3 and 30 *μ*M, respectively (Figure 2C).

### Biodistribution of radiolabelled annexin V

**Figure 3:**
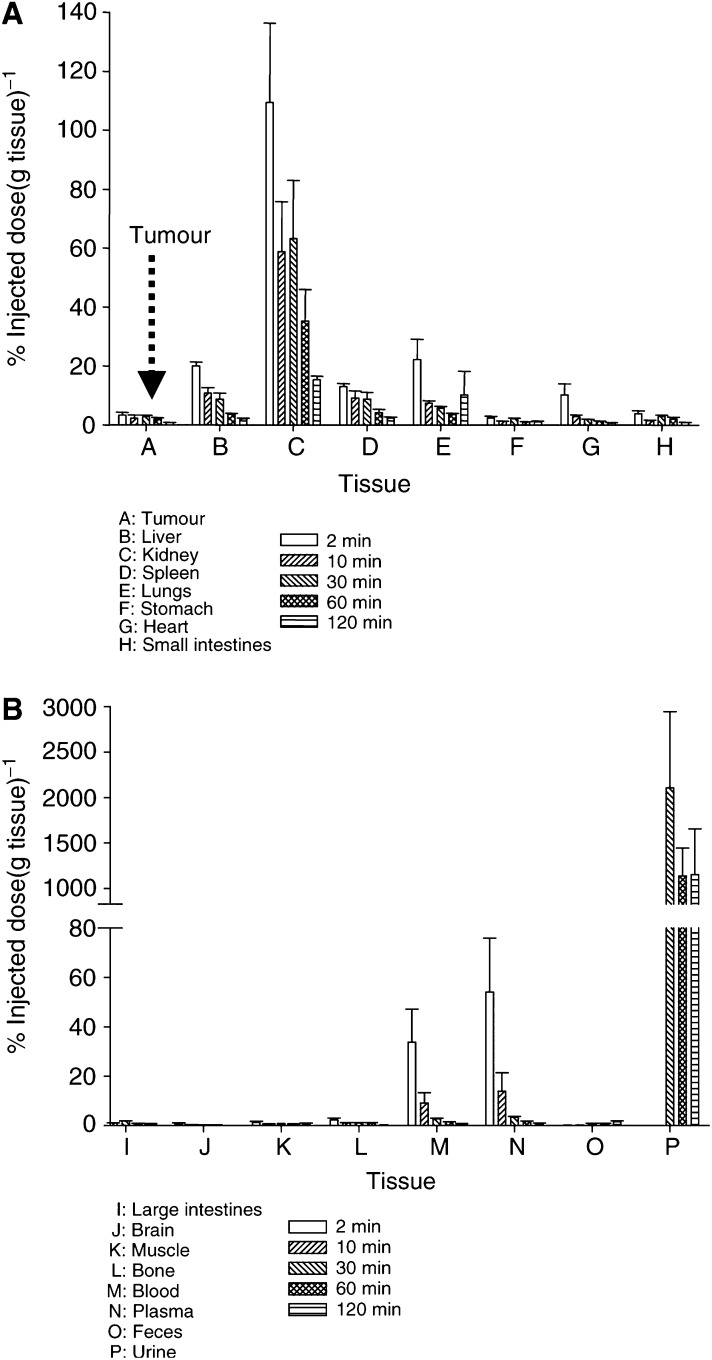
Biodistribution of [^125^I]SIB–annexin V in untreated RIF-1 tumour bearing C3H/Hej mice at 2, 10, 30, 60 and 120 min. Data from (**A**) tumour, liver, kidney, spleen, lungs, stomach, heart and small intestines, and (**B**) large intestines, brain, muscle, bone, blood, plasma, faeces and urine. The two panels show data from the same group of mice; note that the *Y*-axis is different for the two panels. Data are mean±s.e.m. (*n*=3–5 per time point).

The biodistribution of [^125^I]SIB–annexin V in RIF-1 bearing C3H/Hej mice is shown in Figure 3

. [^125^I]SIB–annexin V distributed well into all tissues. The radioactivity profiles in all tissues showed a rapid uptake such that the highest radioactivity was seen at the first time point (2 min). The washout of radioactivity was slower. Overall, the highest tissue radioactivities were seen in kidneys and urine. At early time points, radioactivity in the blood, heart, lungs, spleen and liver was higher than that in untreated tumours, although the clearance from these tissues was more rapid. This observation was important in the selection of 1 h as the optimal time for evaluating apoptosis in tumours.

### Radiolabelled annexin V localises in apoptotic tumours *in vivo*

We hypothesised that the proof of mechanism of action of radiolabelled annexin V *in vivo* will involve a higher uptake in apoptotic tumours compared to control tumours. The 48 h time point had the highest uptake after treatment (data not shown) and, thus, used for subsequent experimentation. In keeping with the above hypothesis, the uptake of indirectly radiolabelled tracer, [^125^I]SIB–annexin V, was found to be 2.3-fold higher in 5-FU-treated tumours (48 h postinjection) compared to control tumours (*P*=0.04; Table 1

**Table 1:** Effect of 5-FU on the tumour uptake of [^125^I]SIB–annexin V and [^125^I]annexin V *in vivo* in control and 5-FU-treated (165 mg kg^−1^ i.p.; 48 h post-treatment) mice

	**[^125^I]SIB–annexin V**	**[^125^I]annexin V**
Control	0.36±0.05^a^	0.94±0.17
5-FU treated	0.84±0.16	0.99±0.05
Challenge	ND	0.55±0.03

In all mice, the radiotracer was injected and biodistribution studies were performed at 60 min. In one group of mice (challenge experiment), 5-FU-treated mice were pretreated with 100 times unlabelled annexin V at 60 min prior to radiotracer injection. 5-FU=5-fluorouracil; SIB=*N*-succinimidyl-3-iodobenzoic acid.

a Data are mean tumour-to-blood ratios±s.e.m. (*n*=3–10).

). In contrast, there was a nonsignificant change (*P*=0.91) in the uptake of the directly radiolabelled tracer, [^125^I]annexin V, in 5-FU-treated tumours compared to control tumours (Table 1). The binding of [^125^I]annexin V to annexin V-binding sites was, however, found to be specific as indicated by the ability to block uptake by pretreatment of mice with excess unlabelled annexin V (*P*=0.04).

**Figure 4:**
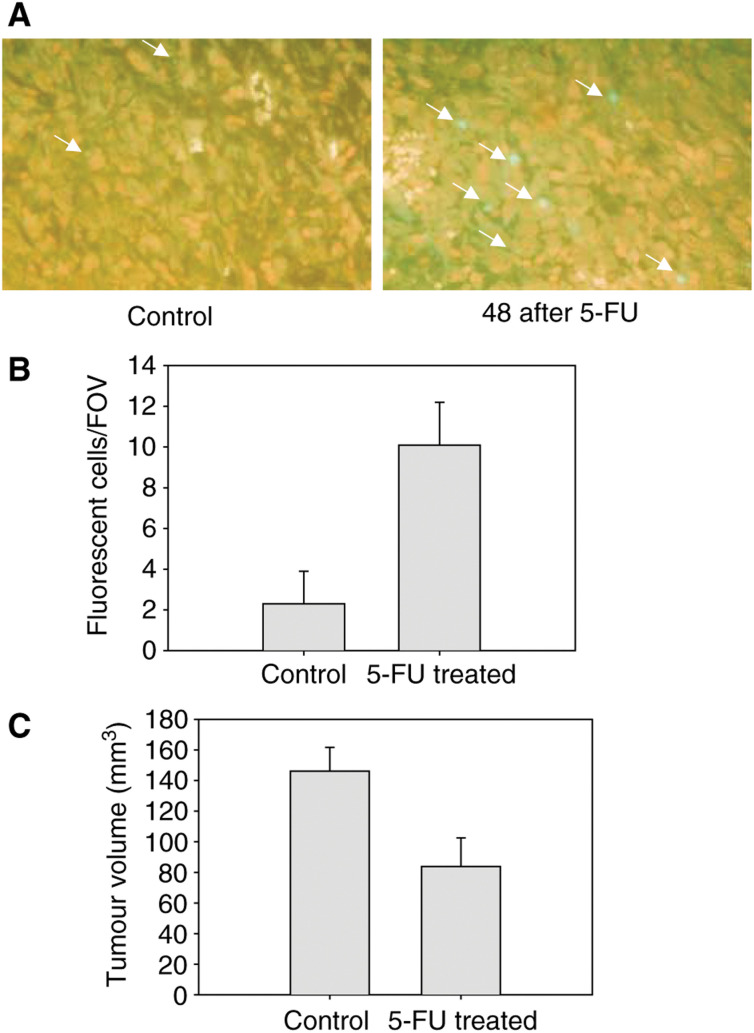
5-Fluorouracil-induced cell death in RIF-1 cells *in vivo*. (**A**) Representative 5 *μ*m immunohistochemical (TUNEL) sections of control and 5-FU treated (165 mg kg^−1^ i.p.; 48 h postinjection) tumours; × 400 magnification. (**B**) The average number of apoptotic cells (TUNEL positive) per field of view (0.55 mm^2^) for control and 5-FU-treated groups. Two slices from three tumours were analysed for each group; for each section, 10 fields of view were randomly selected for the analysis. (**C**) Mean tumour size of pretreated (control) and 5-FU-treated (165 mg kg^−1^ i.p.; 48 h postinjection) mice. There was a 43% reduction of tumour volume. Data are mean±s.e.m. (*n*=6, pretreated; *n*=3, 5-FU-treated).

The radiotracer-binding characteristics were in keeping with TUNEL immunostaining of histological sections. Figure 4A

shows representative TUNEL-stained section of control and 5-FU-treated RIF-1 tumours. TUNEL-stained cells are seen in both sections. The number of TUNEL-positive cells increased by 4.4-fold after 5-FU treatment (*P*=0.0001; Figure 4B). A substantial reduction in tumour volume was seen at 48 h post 5-FU injection (43%; *P*=0.05; Figure 4C).

### Imaging of radiolabelled annexin V in mice by PET

**Figure 5:**
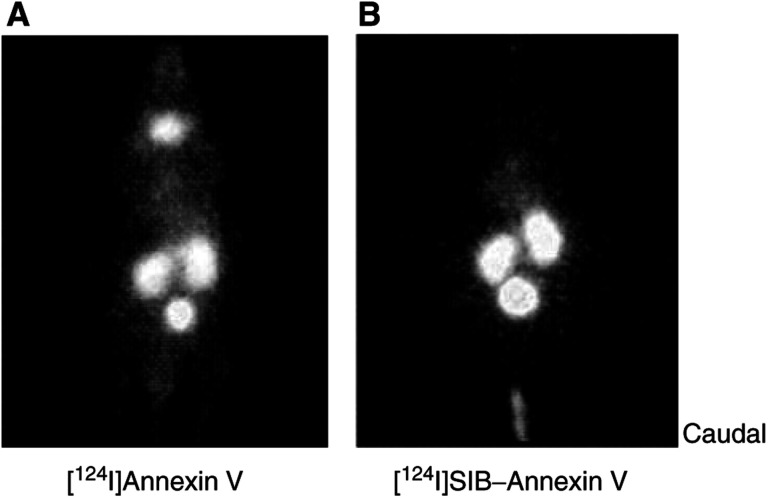
Maximum intensity projection PET images of 5-FU treated (165 mg kg^−1^ i.p.; 48 h postinjection) RIF-1 tumour-bearing C3H/Hej mice injected with (**A**) 40 *μ*Ci of [^124^I]annexin V or (**B**) 10 *μ*Ci [^124^I]SIB–annexin V. All time frames from 30 to 60 min (for all coronal planes) were summed to give an indication of retention. With both radiotracers, there was localisation of radioactivity in the kidneys and bladder; the flank tumours were not visible. In the case of [^124^I]annexin V, there was localisation of radioactivity in the thyroid region. Orientation=tail at the bottom of the image; head at the top.

Positron emission tomography offers the potential of imaging radiolabelled-annexin V binding with high sensitivity and resolution (temporal and spatial). Thus, we imaged 5-FU-treated RIF-1 tumour-bearing mice (48 h post-treatment) with either [^124^I]annexin V or [^124^I]SIB–annexin V in a dedicated small animal PET scanner. Representative 30–60 min maximum intensity projection images are shown in Figure 5

. Due to the low radiochemical yield, the amount of radiotracer injected in both cases was low. This did not permit the full potential of dynamic imaging to be achieved. The static image data were, however, in general agreement with the biodistribution data showing high uptake in the kidney and bladder regions. The main difference between the two radiotracers was a high uptake of radioactivity in the thyroid in the case of [^124^I]annexin V. In this study, we did not detect the tumour by PET imaging.

## DISCUSSION

The experiments described in this paper underpin the potential utility of iodinated annexin V as a radiotracer for imaging apoptosis by PET. Direct and indirect methods of radiolabelling annexin V with radioiodine were employed to synthesise [^125^I]annexin V and [^125^I]SIB–annexin V, respectively. Direct iodination yielded higher pseudospecific activity product than indirect labelling with SIB. *In vitro* analysis demonstrated that the two radiotracers were biologically active, although the absolute proportion of the bound tracer was higher in the case of [^125^I]SIB–annexin V. Although there was qualitative agreement between radiotracer uptake, TUNEL flow cytometry staining and clonogenic survival data in these *in vitro* studies, there were significant quantitative differences. The increase in radiotracer binding between 3 and 30 *μ*M 5-FU was at least 10-fold lower than the corresponding changes in flow cytometry and clonogenic survival data. These differences may be explained by the different phases of the apoptotic process detected by annexin V *vs* TUNEL (Martin *et al*, 1995), as well as the ability to gate out nonspecific effects in the flow cytometry studies. The survival data would suggest that small changes in radiotracer binding could lead to larger changes in clonogenic survival.

Positron emission tomography images of mice showed an absence of radioactivity in the thyroid region with [^125^I]SIB–annexin V, suggesting that the indirect iodination with SIB led to a product that was more stable to deiodination *in vivo*. This finding is in agreement with the work of Russell *et al*, who demonstrated that indirect labelling (Bolton-Hunter) of annexin V gave a product that was more stable to deiodination than [^125^I]annexin V (Russell *et al*, 2002). In theory, SIB should be more stable to deiodination than Bolton-Hunter given the presence of a *para*-hydroxyl group (iodine in *meta*-position) in the latter. Deiodination leads to lower specific labelling of PS, which may explain why a significant increase in labelling was seen with the indirectly radiolabelled annexin V, but not the directly radiolabelled product. Thus, [^124^I]SIB–annexin V appears to be a superior radiotracer for PET imaging of apoptosis *in vivo*, although its radiochemical yield needs to be improved further to enable quantitative kinetic measurements to be performed. Alternatively, the phenyl-containing precursors can be reacted with ^18^F^−^ to form prosthetic groups for the synthesis of 18F-radiolabelled annexin (Vaidyanathan and Zalutsky, 1992). Fluorine-18-radiolabelled annexin will have a higher proportion of photons (∼75% higher) and lower positron energy, thus providing the potential to perform kinetic measurements and improve image quality. The development of other positron-emitting isotopes (^11^C and ^68^Ga) conjugated to annexin V has been reported at recent imaging meetings. With regard to clinical imaging, these other radiotracers may have advantages including lower organ absorbed dose due to their shorter half-lives and higher resolution due to their lower positron energy. Due to its long half-life, however, ^124^I-radiolabelled annexin V can be made widely available to many PET centres.

The *in vivo* models that have been used in the past to study apoptosis by radiolabelled annexin V have largely been noncancer model systems or models that give artificially high levels of apoptosis (Blankenberg *et al*, 1998, 1999, 2001; Hofstra *et al*, 2000; Ogura *et al*, 2000; Narula *et al*, 2001; Kown *et al*, 2002; Russell *et al*, 2002). Here, we tested the feasibility of detecting the relatively small changes (2–6-fold over pretreatment levels; 3–5%) in apoptosis that are usually achievable in animal and human solid tumours following chemotherapy or biological therapy (Aboagye *et al*, 1998; David *et al*, 1999; Griffon-Etienne *et al*, 1999; Herbst *et al*, 2002). We have provided proof of concept for the use of radiolabelled annexin V to detect changes in apoptosis *in vivo* in tumours. Radiotracer uptake was found to be associated with an increase in TUNEL-stained cells, a decrease in clonogenic survival and tumour shrinkage. [^125^I]SIB–annexin V was more discriminatory than [^125^I]annexin V for this purpose. The binding of [^125^I]annexin V to PS could be blocked *in vitro* and *in vivo* by excess of unlabelled annexin V. In either situation, the levels of radiotracer binding in the blocking studies were less than that of control cells. This could be due to the presence of apoptotic cells in the untreated control cells. In this study, we did not detect the tumour by PET imaging. The inability to detect the (flank) tumour by PET may be due to its proximity to the kidney and bladder, which had very high uptake values. Even though *in vivo* [^124^I]SIB–annexin V failed to detect apoptosis in our model system, other experiments employing annexin V indirectly radiolabelled with ^99m^Tc have demonstrated the feasibility to localise cell death in regions distant from the liver and kidneys (Belhocine, 2002).

This technology has a number of limitations that need to be considered in the translation of these preclinical studies into clinical trials: (i) untreated tumours showed low radiotracer uptake (%ID g^−1^) compared to that of the kidney, urine, liver, lung and spleen, and induction of apoptosis did not produce a high image contrast for tumours. Coregistration with magnetic resonance or X-ray computed tomography images may be required to aid tumour definition. (ii) The very high localisation of radioactivity specifically in the kidney and urine may lead to low signal-to-background ratio and ‘spill over’ in the clinical PET imaging of kidney and bladder tumours, which could affect quantification. The high localisation of radiolabelled annexin in the kidney has been reported by other workers (Blankenberg *et al*, 1998, 1999). Although the reason for this nonspecific uptake is not fully understood, it is likely related to the unique phospholipids composition of the renal cortex, which has higher amounts of PS compared to the papillary regions. Another explanation is the nonspecific uptake of low-molecular-weight proteins such as annexin V by the proximal renal tubule cells (Blankenberg *et al*, 1999). (iii) Necrotic cells will be expected to bind to radiolabelled annexin V. In this regard, initial studies have shown that heat treatment of HL60 cells leads to substantial radiotracer binding (DR Collingridge and EO Aboagye, unpublished data). Thus, unless there is prior evidence of low necrotic death following drug treatment, which is the case for 5-FU treatment of RIF-1 tumours *in vivo* (Aboagye *et al*, 1998), changes in radiolabelled annexin V binding should be interpreted as changes in cell death rather than changes in apoptosis. In the clinical development of [^124^I]SIB–annexin V for cancer therapy monitoring, additional measures of viability, for example, the exchanging H_2_^15^O space in tissues by PET (Wilson *et al*, 1992) or T_2_ relaxation maps by MRI (Heywang *et al*, 1987), may be required to differentiate between apoptosis and necrosis. (iv) PET imaging with radiolabelled annexin V provides a ‘snapshot’ of apoptosis. For translation into clinical application, the challenge will be the determination of when to scan (Blankenberg, 2002). In this study, we selected 48 h post-treatment as the time point to image cell death, based on previous studies that indicated a peak in the apoptotic index at this time point (Aboagye *et al*, 1998). More extensive studies are required to show whether this time coincides with peak annexin V binding. Belhocine *et al* (2002) demonstrated an increase in labelling of human tumours with [^99m^Tc]annexin V and SPECT at 24–48 h after the first course of chemotherapy. This agrees with reports by Ellis *et al* (1997), for instance, of increased apoptosis in human breast tumours measured in the biopsy specimen, 24 h after a course of chemotherapy. In general, the pharmacokinetics profile (peak concentration, time to achieve peak concentration, half-life, steady-state concentration and exposure) and mechanism of action (DNA interacting drugs *vs* signal transduction inhibitors, for instance) of administered drugs are important considerations in selection of the optimal time for the post-treatment scan. In addition, serial studies may be required to establish the optimal imaging protocol that will give maximal tumour/normal tissue contrast and signal intensity.

In conclusion, we have provided proof of principle for the use of iodine-labelled annexin V in detecting chemotherapy-induced apoptosis *in vitro* and in tumour-bearing mice. The method provides a ‘snapshot’ of apoptotic cells (and possibly necrotic cells as well). The small changes in radio-annexin binding are associated with larger changes in clonogenic survival or tumour shrinkage. [^124^I]SIB–annexin V in particular, warrant further preclinical and clinical development as a probe for imaging apoptosis induced by chemotherapeutic agents.
